# The Association Between Social Media Usage on Food Choice Motivations and Dietary Carbon Footprints in Adolescents: A Cross-Sectional Study

**DOI:** 10.3390/ijerph23030400

**Published:** 2026-03-21

**Authors:** Hande Seven Avuk, Tugce Ozlu Karahan, Ezgi Sarigil, Nil Pinar, Ayse Terzi, Nursena Dirinli, Emre Batuhan Kenger

**Affiliations:** 1 Department of Nutrition and Dietetics, Faculty of Health Sciences, Istanbul Bilgi University, Istanbul 34440, Türkiye; ezgi.sarigil@bilgiedu.net (E.S.); nil.pinar@bilgiedu.net (N.P.); ayse.terzi@bilgiedu.net (A.T.); nursena.dirinli@bilgiedu.net (N.D.); emre.kenger@bilgi.edu.tr (E.B.K.); 2 Department of Nutrition and Dietetics, Hamidiye Faculty of Health Sciences, University of Health Sciences, Istanbul 34668, Türkiye; tugce.ozlukarahan@sbu.edu.tr

**Keywords:** Adolescent, carbon footprint, food choice, social media, sustainable nutrition

## Abstract

**Highlights:**

**Public health relevance—How does this work relate to a public health issue?**

- This study addresses the intersecting public health topics of adolescent nutrition and environmental sustainability by exploring the potential associations between digital media use and dietary carbon footprints.
- It highlights social media as a relevant digital environment that may relate to adolescents’ food choice motivations and their everyday dietary behaviors.

**Public health significance—Why is this work of significance to public health?**

- The findings suggest a modest inverse association between health-oriented food choices and dietary carbon footprints, aligning with the “One Health” framework.
- The research indicates that usage of different social media platforms is associated with varying food choice motivations, such as weight control or ethical concerns, particularly among female adolescents.

**Public health implications—What are the key implications or messages for practitioners, policy makers and/or researchers in public health?**

- Public health practitioners could consider the role of social media platforms when developing nutrition education strategies aimed at promoting both personal and planetary health.
- Policymakers and dietitians should be mindful that interventions encouraging sustainable, lower-carbon diets require careful planning to prevent potential nutrient inadequacies during adolescent development.

**Abstract:**

Social media has become a prominent digital environment associated with adolescents’ food preferences and the environmental impacts of their diets. This study aimed to examine the relationship between social media usage habits, food choice motivations, and the environmental impact of the diet, specifically the carbon footprint, in adolescents. This cross-sectional study was conducted with 216 adolescents aged 14–18 years in Istanbul between January and April 2025. Data were collected using the Food Choice Questionnaire (FCQ) and a 24 h dietary recall. The dietary carbon footprint was calculated by mapping 24 h dietary recall data to emission factors from the Data FIELDS database and scientific literature. Of the participants, 60.6% were female. Females had significantly higher rates of being influenced by social media in food choices (*p* < 0.001) and total FCQ scores (*p* = 0.025) compared to males. Regarding social media platforms, TikTok usage was associated with higher ethical concern and mood scores (*p* < 0.001), while Instagram usage was associated with weight control (*p* = 0.012). Daily internet use of 180 min was associated with higher price (*p* = 0.001) and weight control (*p* = 0.003) motivations. Notably, a significant negative correlation was found between health motivation and carbon footprint (r = −0.173, *p* = 0.011). Multivariate regression analysis confirmed that an increase in health score was associated with a reduction in carbon footprint (β = −0.204, *p* = 0.003), independent of gender, BMI, and social media influence. Social media platforms serve as a relevant digital environment associated with adolescents’ food preferences. The finding that health-oriented choices are associated with lower carbon footprints indicates that promoting healthy eating on social media will benefit both individual and planetary health.

## 1. Introduction

The food system has been identified as a major contributor to climate change, and the primary sources of greenhouse gas emissions are nitrous oxide from soils, methane from enteric fermentation in animals, and carbon dioxide resulting from land-use changes such as deforestation [1]. Water and carbon footprint calculation methods are employed to determine the environmental impacts of foods and dietary patterns. Foods with lower water and carbon footprints have a reduced environmental impact. The carbon footprint of a food product is the total greenhouse gas emissions generated throughout its production, transportation, storage, processing, packaging, preparation, and consumption. Meat products have a significantly higher carbon footprint compared to grain or vegetable products [2,3]. Furthermore, the intersection of individual food choices and environmental impact has become a central theme in public health. Incorporating sustainability as a primary motive during food purchasing has been strongly associated with adopting healthier overall dietary patterns [4]. Indeed, individual dietary choices have a profound impact on both personal health and the environmental footprint, demonstrating that healthy, plant-based food choices inherently converge with environmentally beneficial outcomes [5].

As a prominent digital environment, social media usage is strongly associated with individuals’ food preferences [6]. Health awareness campaigns on social media play a significant role in shaping user behavior. For instance, food-related content shared on platforms such as Twitter, YouTube, and Instagram may reflect broader dietary trends in society. Research has demonstrated that food-related tweets shared by users are associated with obesity rates [7]. Social media has evolved into a platform that influences individuals’ sustainability awareness and behaviors in a multifaceted manner. Social media, where topics ranging from recipes to eco-friendly dietary habits are disseminated, can shape the understanding of sustainability at both individual and societal levels [8]. Ethical and healthy food content deliberately disseminated on social media may enable individuals to make more conscious, sustainable choices in the long term [9].

The theoretical mechanism linking this social media exposure to diet-related carbon footprints primarily operates through the mediating role of food choice motivations. As Simeone and Scarpato [10] emphasize, the relationship between social media information and sustainable consumption is complex. While digital platforms can significantly increase environmental awareness, they can also inadvertently promote unsustainable food behaviors, depending on the information sources users follow. As a supporting example, a recent bibliometric review by Coman et al. [11] reveals that platforms emphasizing visual narratives create a digital peer effect that functions as a significant tool in shaping individuals’ ethical and sustainable nutrition decisions. Furthermore, in their study focusing on Generation Z, Confetto et al. [12] demonstrated that exposure to social media content acts as a powerful stimulus for young people, directly triggering sustainable consumption behaviors and habits.

Although the general effects of social media on nutrition have been examined in the existing literature, studies investigating how this digital exposure translates into tangible carbon footprint outcomes through specific motivations are quite limited. In light of this information, this study aims to contribute to the literature by examining the potential associations between adolescents’ social media usage habits and their food choice behaviors and dietary carbon footprints.

## 2. Materials and Methods

### 2.1. Study Design and Participants

**Figure 1:**
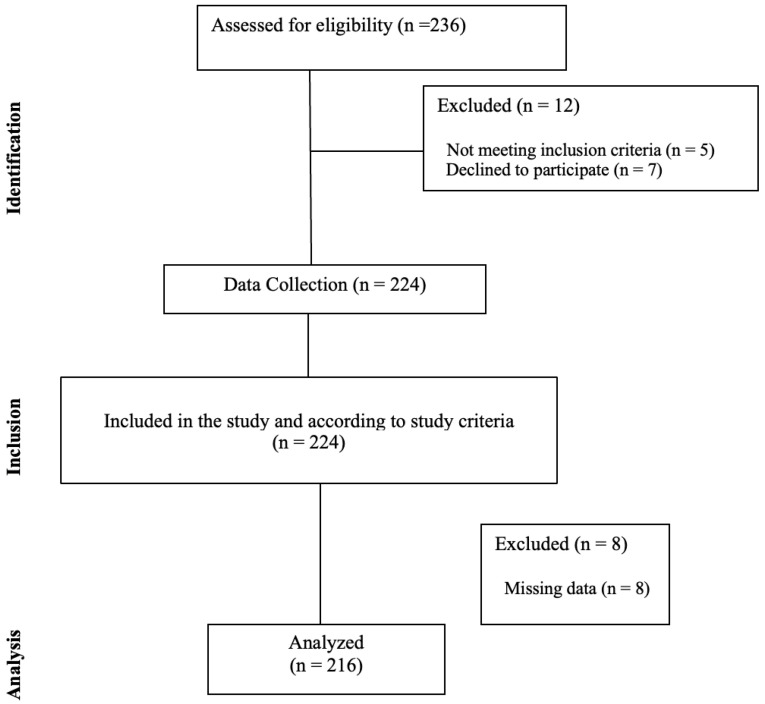
STROBE flow diagram of the study.

This cross-sectional study was conducted between January and April 2025 with 216 adolescents aged 14–18 attending two private high schools in Istanbul. Participants were recruited using a convenience sampling method. The sample size was determined using a priori power analysis via G*Power 3.1.9.4 software [13]. Based on a medium effect size, a 95% confidence interval (alpha = 0.05), and 95% power (1−β = 0.95), the minimum sample size required for the study was calculated as 216. Adolescents falling outside the 14–18 age range, following a specific medical diet, declining to participate, or providing incomplete data were excluded from the study. During the recruitment phase, a total of 236 potential participants were initially assessed for eligibility. Adolescents falling outside the 14–18 age range or following a specific medical diet were defined as not meeting the inclusion criteria (n = 5). Additionally, 7 adolescents declined to participate, resulting in 12 initial exclusions. Data were collected from the remaining 224 students; however, 8 participants were subsequently excluded from the final analysis due to providing incomplete or missing data. Consequently, the final sample consisted of 216 adolescents. The detailed STROBE flow diagram of the study is presented in Figure 1.

Ethical approval for the study was obtained from the Istanbul Bilgi University Human Research Ethics Committee in accordance with the Declaration of Helsinki (Approval No: 2024–10160-208, Date: 20 December 2024). Prior to data collection, researchers visited classrooms to inform potential participants about the study’s objectives and procedures. During these sessions, the questionnaire content and necessary information were outlined, with strong emphasis on the voluntary nature of participation. Students were requested to obtain written informed consent from their parents. The participant recruitment process was completed upon the collection of these signed consent forms.

### 2.2. Data Collection Tools

The questionnaires were administered face-to-face, and researchers provided guidance in the classroom throughout the process to avoid any potential confusion. The research instrument consisted of three sections: sociodemographic data and anthropometric measurements, the Food Choice Questionnaire (FCQ), and a 24 h dietary recall. Based on the collected data, daily dietary carbon footprints were calculated for each participant.

Survey Form: Information on adolescents’ age, gender, education level, dietary habits, social media use, and regular physical activity status was collected through the survey form.

Anthropometric Measurements: Participants’ heights were measured while standing barefoot on a flat surface, with their heads in the Frankfort horizontal plane [14]. Body weight was measured and recorded to the nearest 50 g using a TANITA BC-601 portable digital body composition monitor, with participants wearing light clothing and no shoes. Body Mass Index (BMI) was calculated by dividing body weight by the square of height (meters) [15].

Food Choice Questionnaire (FCQ): The FCQ is a 36-item questionnaire comprising 9 sub-dimensions that assess intrinsic and extrinsic food attributes that motivate food choices. The scale was initially developed by Steptoe et al. [16], and its validity and reliability for the Turkish population were established by Dikmen et al. [17]. The sub-dimensions of the scale are health, mood, convenience, sensory appeal, natural content, price, weight control, familiarity, and ethical concern. In the FCQ, participants are asked to rate the importance of each item for their food choices on a typical day using a 4-point Likert scale (1 = “not at all important”, 2 = “a little important”, 3 = “moderately important”, 4 = “very important”)—the intraclass correlation coefficients (ICCs) of the original scale range from 0.89 to 0.95.

Dietary Assessment: Participants’ dietary intake was evaluated using a 24 h dietary recall conducted for a random weekday. The Nutrition Information Systems (BeBiS) software (Full version 8.2) was used to calculate the individuals’ macro- and micronutrient intake levels.

Calculation of Carbon Footprint: A comprehensive national Life Cycle Assessment (LCA) database reflecting the environmental impacts of foods specific to Turkey is not yet available. Therefore, this study used the “Data FIELDS” (Food Impacts on the Environment for Linking to Diets) database [18], which presents the environmental impacts of foods in standardized categories and is based on life-cycle assessment literature. Although absolute emission values vary depending on geographical conditions, it was assumed that the proportional differences between food groups (e.g., animal sources having significantly higher emissions than plant sources) are globally consistent. Thus, this database was accepted as a valid tool for comparing dietary patterns. In addition to the database, current average carbon footprint values published in the scientific literature were also utilized for the calculations [19,20,21]. To ensure methodological transparency, mixed or composite dishes reported in the 24 h dietary recalls were systematically disaggregated into their individual raw ingredients using a national standard recipe reference book [22]. For mixed dishes or specific local foods not included in this reference, detailed information regarding specific ingredients and preparation methods was obtained directly from participants during the dietary recall interviews. These specific items were then correlated with commonly used standard recipes. Subsequently, all individual ingredients and their consumed quantities were manually matched to the closest corresponding food items within the Data FIELDS database to apply the appropriate emission factors. The emission factors, defined in the literature per kilogram (kg CO_2_-eq/kg product), were applied to the portion sizes from participants’ 24 h dietary recalls and converted to grams (g CO_2_-eq/g product). To evaluate the diet’s total environmental impact independent of energy intake, participants’ carbon footprint values were standardized and calculated per 1000 kcal. Spices and other flavorings present at trace levels were excluded from the analysis due to insufficient emission factors and their negligible contribution to the total carbon footprint.

### 2.3. Statistical Analysis

The data obtained in the study were analyzed using SPSS (Statistical Package for the Social Sciences) for Windows, version 30.0. Normally distributed data were expressed as mean ± standard deviation, while non-normally distributed data were expressed as median (25th–75th percentiles). Categorical variables were presented as counts and percentages. Data distribution normality was assessed using the Kolmogorov–Smirnov test. For comparisons between two groups, the independent-samples t-test was used for normally distributed continuous variables, and the Mann–Whitney U test was used for non-normally distributed continuous variables. The relationships between categorical variables were evaluated using the Chi-square test. The relationships between carbon footprint per energy and energy/nutrient intake were examined using Spearman’s correlation analysis. Multivariate linear regression analysis was performed to evaluate the independent association of the health sub-dimension of the Food Choice Questionnaire with carbon footprint. Specifically, to avoid unnecessary model complexity and reduce the risk of overfitting, only the FCQ health subscale, which showed a statistically significant correlation with carbon footprint in the preliminary analysis, was included in the multivariate regression model. Prior to the multivariate linear regression analysis, the carbon footprint variable was logarithmically transformed to approximate normality. Prior to the multivariate linear regression analyses, model assumptions were evaluated. Residuals were approximately normally distributed based on histograms and P–P plots. Scatterplots of standardized residuals versus predicted values indicated no heteroscedasticity. Multicollinearity was not detected, as variance inflation factor (VIF) values were close to 1. Following the crude model, adjustments were made sequentially for gender (Model 1), body mass index (Model 2), the influence of social media on food choice (Model 3), and internet usage status (Model 4). Regression results were reported as standardized beta coefficients, 95% confidence intervals, and *p*-values. Statistical significance was set at *p* < 0.05 for all analyses.

## 3. Results

The study sample consisted of 216 adolescents with a mean age of 16.9 ± 0.94 years (60.6% female; 39.4% male). When general characteristics were examined, 63.9% of those engaging in regular physical activity were male, whereas 70.3% of those who did not were female (*p* < 0.001). Of those who believed social media influenced their food choices, 72.0% were female, while only 28.0% were male (*p* < 0.001). Similarly, the vast majority (65.9%) of those stating that food advertisements on social media triggered hunger were female (*p* = 0.011). The rates of following dietitians and reading nutrition-related articles were significantly higher in females than in males (*p* < 0.05; Supplementary Table S1). Carbon footprint tertiles did not differ significantly between genders (*p* = 0.532). In the Food Choice Questionnaire (FCQ), the total score was significantly higher for females compared to males (96.9 ± 17.2 vs. 91.8 ± 20.7; *p* = 0.025). Specifically, female scores were significantly higher in the sub-dimensions of mood (17.1 ± 4.3 vs. 15.2 ± 4.8; *p* = 0.002), sensory appeal (13.0 ± 2.3 vs. 12.1 ± 2.8; *p* = 0.007), natural content (6.8 ± 2.4 vs. 6.1 ± 2.3; *p* = 0.031), ethical concern (6.9 ± 2.5 vs. 6.2 ± 2.7; *p* = 0.045), and weight control (6.9 ± 2.9 vs. 6.2 ± 2.5; *p* = 0.033). Carbon footprint tertiles did not differ significantly between genders (*p* = 0.532) (Table 1).

When adolescents’ food choices and carbon footprints were evaluated by internet usage, the total food choice score was significantly higher in individuals with daily internet use of ≥180 min than in those with <180 min (97.8 ± 18.5 vs. 93.2 ± 18.8; *p* = 0.042). In this group, the scores for price (9.0 ± 2.4 vs. 7.9 ± 2.7; *p* = 0.001) and weight control (7.4 ± 3.0 vs. 6.3 ± 2.6; *p* = 0.003) sub-dimensions were significantly higher. The total food choice score of TikTok users was higher than that of non-users (99.1 ± 19.8 vs. 91.6 ± 17.4; *p* = 0.002). Additionally, the health (16.0 ± 4.3 vs. 14.3 ± 4.8; *p* = 0.005), mood (17.9 ± 4.6 vs. 15.1 ± 4.3; *p* < 0.001), and ethical concern (7.2 ± 2.7 vs. 6.1 ± 2.5; *p* < 0.001) sub-dimensions were significantly higher in TikTok users. Among Instagram users, health (15.7 ± 4.7 vs. 13.9 ± 4.5; *p* = 0.004), weight control (7.0 ± 2.7 vs. 6.1 ± 2.8; *p* = 0.012), and total food choice scores (96.5 ± 19.3 vs. 91.9 ± 17.6; *p* = 0.042) were found to be higher. In individuals using X, the price score was significantly higher (9.8 ± 2.0 vs. 8.2 ± 2.6; *p* = 0.018) (Table 2).

**Figure 2:**
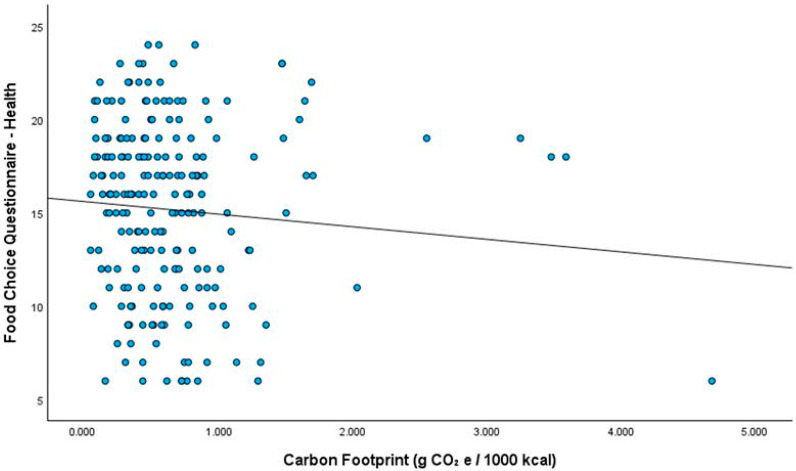
Relationship between health subscale score of food choice questionnaire and carbon footprint.

A weak but statistically significant negative correlation was found between the carbon footprint and the health sub-dimension of the Food Choice Questionnaire (r = −0.173; *p* = 0.011) (Figure 2). In contrast, no significant relationship was observed between the other sub-dimensions of food choice and the carbon footprint per 1000 kcal (*p* > 0.05; Supplementary Table S2). In the multivariate linear regression analysis examining the association between the health sub-dimension of the Food Choice Questionnaire and carbon footprint, a negative, statistically significant relationship was observed between the health score and carbon footprint. In the crude model, the health score was inversely associated with the carbon footprint (β = −0.197; *p* = 0.004). This relationship remained significant in Model 1 adjusted for gender (β = −0.185; *p* = 0.006), Model 2 adjusted for gender and BMI (β = −0.191; *p* = 0.005), Model 3 adjusted for gender, BMI, and the influence of social media on food choice (β =−0.204; *p* = 0.003), and Model 4 adjusted for gender, BMI, the influence of social media on food choice, and internet usage status (β = −0.204; *p* = 0.003). The inclusion of internet usage in the model did not alter the coefficient or the direction of the relationship (Table 3).

## 4. Discussion

This study aimed to examine the relationship between social media usage habits, food choice motivations, and the dietary carbon footprint—an indicator of the environmental impact of diet—among adolescents. The findings reveal that social media, and particularly health perception, are significantly associated with sustainable dietary behaviors.

Adolescents’ dietary behaviors cannot be considered independently of their physical and social environments. In a study examining the factors affecting food intake in children and adolescents, Gubbels [23] emphasizes that the media creates a ‘food environment’ that is just as determinant as the physical environment. The significant association between social media (TikTok, Instagram) and food choice motivations observed in our study represents the digital equivalent of this environmental determinant concept highlighted by Gubbels [23]. Through the visual and auditory stimuli it provides, social media exposure is linked to variations in what adolescents eat but also the meanings they attribute to food, such as ethics, mood, and health.

In our study, it was observed that female participants were significantly more influenced by social media compared to males, followed dietitians at a higher rate, and exhibited significantly higher scores in the weight control, mood, and natural content sub-dimensions. This finding aligns with the literature indicating that females are more susceptible to health and body-image messages [5,24]. Indeed, current research reports increased body dissatisfaction and eating disorder symptoms among females who follow nutrition-focused influencers on Instagram [25,26,27,28]. In our study, the high weight control scores observed in females can be interpreted as a reflection of social media’s impact on body image.

When examined on a platform basis, the association appears to vary. The high scores for health and ethical concerns among TikTok users suggest that this group may possess the potential for mindful eating. In fact, the literature indicates that positive and mindful approaches to eating are associated with healthy food choices [29] and can shape sustainable dietary behaviors [30]. Among Instagram users, the high motivation for ‘weight control’ aligns with the platform’s visually visual-oriented nature and the risk of Orthorexia Nervosa [31]. Furthermore, the increase in price and weight control scores observed as daily internet usage exceeds 180 min highlights the paradoxical association of digital exposure. The literature emphasizes that this relationship is neither unidimensional nor linear. Indeed, influencer interactions can sometimes guide individuals toward healthy choices through a prevention-focused motivation [32], while at other times they may harm psychological well-being, even when dietary quality improves [33]. However, the nature of the exposed content plays a determinant role and may pave the way for positive behavioral transformations in long-term interactions [34].

A notable finding of our study is that as the health motivation in food choice increases, the dietary carbon footprint decreases. However, it is crucial to emphasize that while this relationship is statistically significant, the correlation and regression coefficients indicate a modest association. This result supports the “One Health” approach and the Barilla Double Pyramid model, which holds that healthy eating and eco-friendly nutrition support each other [35]. The principle that plant-based foods recommended for health have lower carbon emissions is further supported by correlations between healthy, sustainable behaviors and young adults [36]. While health motivations may lead adolescents to lower-carbon, plant-based options, food choice remains a highly complex process driven by many interacting factors, and health motivation alone may not lead to significant reductions in individual carbon footprints. A study conducted in Turkey also emphasizes that increasing food literacy guides individuals toward conscious and sustainable consumption [37].

The importance of this study becomes clearer when trends across Türkiye are considered. The trend analysis by Ilhan and Rakıcıoğlu [38], based on Turkey Nutrition and Health Survey (TBSA) data, indicates that Türkiye’s carbon footprint increased by 16.1% between 2010 and 2017, driven by red meat consumption, while fruit and vegetable consumption declined. While the environmental impact improved over time in Germany [39], the negative trend in Türkiye underscores the importance of conducting national studies. Furthermore, the significantly higher carbon footprint of males in national data [40] supports our finding that females tend toward lower-carbon choices driven by health and natural content motivations. Adolescents shifting toward plant-based diets due to health concerns could be an effective strategy to reverse this negative environmental trend in Türkiye. However, the association observed in this cross-sectional study does not necessarily mean that promoting healthy eating on social media will directly translate into more sustainable diets in practice. Determining the true effectiveness of such digital interventions is quite complex and requires rigorous evaluation through future experimental research.

Although the carbon footprint-reducing effect of health motivation is promising, the literature reminds us that this relationship must be carefully managed to ensure nutrient adequacy. The simulation study by Temme et al. [41] revealed that environmentally sustainable diets, while improving health, may pose risks regarding the intake of critical micronutrients such as iron and zinc. Similarly, van de Locht et al. [39] reported that environmental sustainability may not always go hand in hand with nutrient adequacy. Therefore, strict carbon footprint reduction targets should be set without disregarding adolescents’ developmental needs.

Several limitations should be considered when evaluating the results of this study. First, the cross-sectional design of the study prevents establishing causal relationships between variables, and the findings reflect the situation only within a specific timeframe. Second, participants were recruited using a convenience sampling method limited to two private high schools in Istanbul. Because the sample was drawn solely from private institutions, it represents a socioeconomically homogeneous group, mostly from the middle and upper income brackets. This homogeneity naturally minimizes significant socioeconomic confounding in our dataset, while the lack of a measure of socioeconomic status prevents us from analyzing the effects of income disparities on the carbon footprint of food-related activities. As this sampling approach does not ensure adequate representativeness of the broader adolescent population in Türkiye, particularly regarding socioeconomic and socio-cultural diversity, it significantly limits the generalizability of our findings. Third, while a single 24 h dietary recall is a widely accepted and practical tool in nutritional epidemiology, a single recall provides a snapshot that may not fully reflect daily or seasonal variations. Although multiple non-consecutive recalls are the ideal standard, they are often difficult to implement in adolescent populations due to high participant load and poor compliance. Additionally, the reported daily energy intakes in our study were relatively low for adolescents aged 14–18 years, which suggests a potential issue of underreporting. Furthermore, the assessment of social media influence relies on self-reported categorical responses; this may not fully capture the nuanced complexity of digital exposure. Finally, the use of the internationally valid “Data FIELDS” database and average values from the literature, due to the lack of a comprehensive food carbon footprint database specific to Turkey, may have led to specific emission differences arising from local production and logistics conditions not being fully reflected in the analyses.

## 5. Conclusions

In conclusion, the findings of this cross-sectional study suggest that social media use among adolescents is associated with different food choice motivations depending on the platform. While causal relationships cannot be established, the modest carbon footprint-reducing associations of health-focused dietary choices highlights a potential shared benefit in public health interventions. However, when developing sustainable nutrition strategies, careful planning is required to prevent nutrient deficiencies. In the future of nutrition, there is a need for holistic education models based on sustainability that do not separate individual health from planetary health. Increasing sustainable and conscious nutrition content on social media platforms has the potential to serve as a tool in combating both obesity and climate change; however, future longitudinal and experimental studies are necessary to verify whether such digital promotions effectively lead to tangible behavioral changes.

## Figures and Tables

**Table 1:** General characteristics of participants.

Characteristics	Female (*n* = 131)	Male (*n* = 85)	Total (*n* = 216)	*p*-Value
Age (years)	17.0 (17.0–17.0)	17.0 (16.0–17.0)	17.0 (17.0–17.0)	**0.031**
Body weight (kg)	57.0 (50.0–62.0)	75.0 (67.0–87.0)	61.0 (55.0–70.0)	**<0.001**
Body mass index (kg/m^2^)	21.1 ± 2.7	23.7 ± 3.8	22.1 ± 3.4	**<0.001**
Regular physical activity				**<0.001**
Yes	22 (36.1)	39 (63.9)	61 (28.2)	
No	109 (70.3)	46 (29.7)	155 (71.8)	
Internet usage (daily)				0.891
<60 min	17 (56.7)	13 (43.3)	30 (13.9)	
60–180 min	65 (61.3)	41 (38.7)	106 (49.1)	
≥180 min	49 (61.3)	31 (38.7)	80 (37.0)	
FCQ				
Health	15.5 ± 4.4	14.5 ± 5.0	15.1 ± 4.7	0.068
Mood	17.1 ± 4.3	15.2 ± 4.8	16.4 ± 4.6	**0.002**
Convenience	14.4 ± 3.4	14.4 ± 3.7	14.5 ± 3.5	0.497
Sensory Appeal	13.0 ± 2.3	12.1 ± 2.8	12.7 ± 2.5	**0.007**
Natural Content	6.8 ± 2.4	6.1 ± 2.3	6.5 ± 2.4	**0.031**
Price	8.1 ± 2.6	8.6 ± 2.6	8.3 ± 2.6	0.064
Weight Control	6.9 ± 2.9	6.2 ± 2.5	6.7 ± 2.8	**0.033**
Familiar	8.1 ± 2.2	8.0 ± 2.2	8.1 ± 2.2	0.478
Ethical Concern	6.9 ± 2.5	6.2 ± 2.7	6.6 ± 2.6	**0.045**
Total FCQ	96.9 ± 17.2	91.8 ± 20.7	94.9 ± 18.8	**0.025**
Carbon Footprint (g CO_2_ e/1000 kcal)	0.42 (0.26–0.66)	0.46 (0.33–0.71)	0.45 (0.30–0.68)	0.092
Carbon Footprint Tertiles				0.532
T1	47 (65.3)	25 (34.7)	72 (33.3)	
T2	41 (56.2)	32 (43.8)	73 (33.8)	
T3	43 (60.6)	28 (39.4)	70 (32.4)	
Energy and Nutrients				
Energy (kcal/day)	1027.2 (764.7–1350.7)	1402.7 (1067.9–1712.7)	1131.2 (885.6–1508.3)	**<0.001**
Carbohydrate (%)	46.0 (40.0–53.0)	46.0 (40.5–54.5)	46.0 (40.0–54.0)	0.419
Protein (%)	15.0 (12.0–19.0)	17.0 (13.5–20.0)	16.0 (12.3–19.0)	**0.008**
Fat (%)	38.1 ± 8.1	34.9 ± 9.7	36.7 ± 8.9	**0.005**
Fiber (g)	10.1 (6.7–13.0)	11.2 (8.1–15.6)	10.5 (7.1–14.7)	**0.016**
Folate (mcg)	133.0 (91.6–194.3)	166.1 (97.4–237.8)	149.5 (92.8–216.1)	**0.031**
Vitamin B_12_ (mcg)	1.6 (0.8–3.2)	2.6 (1.5–5.1)	1.9 (1.0–4.2)	**<0.001**
Vitamin C (mg)	41.3 (22.3–81.5)	35.8 (16.4–62.0)	39.8 (20.0–72.2)	**0.152**
Potassium (mg)	1354.4 ± 600.3	1584.4 ± 825.3	1444.9 ± 704.8	**0.009**
Calcium (mg)	323.1 (216.2–489.5)	397.0 (243.7–540.8)	346.9 (227.1–512.2)	**0.026**
Magnesium (mg)	148.4 (93.9–185.6)	178.2 (121.9–217.7)	157.3 (104.4–198.4)	**0.001**
Zinc (mg)	4.9 (3.5–7.4)	7.2 (4.9–10.2)	5.6 (4.1–8.7)	**<0.001**
Iron (mg)	5.9 ± 2.4	7.7 ± 3.6	6.6 ± 3.1	**<0.001**

Continuous variables showing a normal distribution are expressed as mean ± standard deviation; those not showing a normal distribution are expressed as median (25th–75th percentile); categorical variables are expressed as number (percentage). Independent samples T-test or Mann–Whitney U test was used for continuous variables, and Chi-square test was used for categorical variables. Statistical significance is *p* < 0.05 and is indicated in bold. Abbreviation: FCQ; Food Choice Questionnaire, T; Tertile.

**Table 2:** Food choice questionnaire and carbon footprint values of adolescents according to their daily internet and social media platform usage.

	Internet Usage			TikTok			Instagram			X (Formerly Twitter)		
FCQ	<180 min (*n* = 136)	≥180 min (*n* = 80)	*p*-Value	Yes (*n* = 94)	No (*n* = 122)	*p*-Value	Yes (*n* = 142)	No (*n* = 74)	*p*-Value	Yes (*n* = 13)	No (*n* = 203)	*p*-Value
Health	14.9 ± 4.7	15.3 ± 4.5	0.271	16.0 ± 4.3	14.3 ± 4.8	**0.005**	15.7 ± 4.7	13.9 ± 4.5	**0.004**	14.6 ± 5.8	15.1 ± 4.6	0.345
Mood	16.1 ± 4.6	16.9 ± 4.6	0.086	17.9 ± 4.6	15.1 ± 4.3	**<0.001**	16.6 ± 4.5	15.8 ± 4.7	0.112	15.8 ± 5.7	16.4 ± 4.5	0.331
Convenience	14.2 ± 3.5	14.7 ± 3.5	0.142	14.5 ± 3.5	14.4 ± 3.5	0.412	14.6 ± 3.6	14.2 ± 3.3	0.197	15.8 ± 4.7	14.3 ± 3.4	0.072
Sensory Appeal	12.8 ± 2.6	12.6 ± 2.5	0.353	12.8 ± 2.8	12.5 ± 2.3	0.194	12.8 ± 2.5	12.3 ± 2.5	0.094	12.7 ± 3.1	12.6 ± 2.5	0.461
Natural Content	6.4 ± 2.4	6.7 ± 2.4	0.195	6.6 ± 2.4	6.5 ± 2.4	0.332	6.6 ± 2.4	6.3 ± 2.3	0.173	6.8 ± 2.0	6.5 ± 2.4	0.306
Price	7.9 ± 2.7	9.0 ± 2.4	**0.001**	8.5 ± 2.8	8.2 ± 2.5	0.218	8.2 ± 2.6	8.5 ± 2.7	0.196	9.8 ± 2.0	8.2 ± 2.6	**0.018**
Weight Control	6.3 ± 2.6	7.4 ± 3.0	**0.003**	7.0 ± 2.9	6.5 ± 2.7	0.078	7.0 ± 2.7	6.1 ± 2.8	**0.012**	6.5 ± 2.8	6.7 ± 2.8	0.378
Familiar	8.1 ± 2.2	8.0 ± 2.2	0.388	8.3 ± 2.3	7.8 ± 2.1	0.052	8.0 ± 2.3	8.1 ± 2.1	0.378	8.5 ± 2.4	8.0 ± 2.2	0.258
Ethical Concern	6.4 ± 2.5	6.9 ± 2.7	0.112	7.2 ± 2.7	6.1 ± 2.5	**<0.001**	6.7 ± 2.6	6.5 ± 2.7	0.272	6.9 ± 2.6	6.6 ± 2.7	0.346
Total FCQ	93.2 ± 18.8	97.8 ± 18.5	**0.042**	99.1 ± 19.8	91.6 ± 17.4	**0.002**	96.5 ± 19.3	91.9 ± 17.6	**0.042**	97.6 ± 20.5	94.7 ± 18.7	0.298
Carbon Footprint (g CO_2_ e/1000 kcal)	0.42 (0.31–0.66)	0.49 (0.28–0.70)	0.397	0.46 (0.32–0.67)	0.44 (0.29–0.68)	0.561	0.43 (0.28–0.67)	0.46 (0.33–0.73)	0.197	0.59 (0.28–0.78)	0.44 (0.31–0.67)	0.339

Continuous variables showing a normal distribution are expressed as mean ± standard deviation; those not showing a normal distribution are expressed as median (25th–75th percentile). Independent samples T-test or Mann–Whitney U test was used. Statistical significance is *p* < 0.05 and is indicated in bold. Abbreviation: FCQ; Food Choice Questionnaire.

**Table 3:** Multivariate linear regression models examining the association of the health subscale score of the food choice questionnaire with carbon footprint.

		Carbon Footprint (g CO_2_ e/1000 kcal)				
		β	T	95% Confidence Interval		*p*-Value
				Lower	Upper	
FCQ-Health Score	Crude	−0.197	−2.933	−0.329	−0.064	**0.004**
	Model 1	−0.185	−2.753	−0.317	−0.052	**0.006**
	Model 2	−0.191	−2.841	−0.324	−0.059	**0.005**
	Model 3	−0.204	−3.035	−0.336	−0.071	**0.003**
	Model 4	−0.204	−3.029	−0.336	−0.071	**0.003**

Model 1: Adjusted for gender (R2 = 0.053; *p* = 0.003). Model 2: Adjusted for gender and BMI (R2 = 0.058; *p* = 0.005). Model 3: Adjusted for gender, BMI, and the influence of social media on food choice (R2 = 0.075; *p* = 0.002). Model 4: Adjusted for gender, BMI, the influence of social media on food choice, and internet usage status (R2 = 0.075; *p* = 0.005). Bold values indicate statistical significance. Abbreviation: FCQ; Food Choice Questionnaire.

## Data Availability

The data that support the findings of this study are available from the corresponding author upon reasonable request.

## Supplementary Materials

The following supporting information can be downloaded at: https://www.mdpi.com/article/10.3390/ijerph23030400/s1, Table S1: Nutrition-related social media habits and perceptions of adolescents by gender; Table S2: Relationship between carbon footprint per energy unit and food choice questionnaire scores in adolescents.
