# Supplementary material for: The Association Between Social Media Usage on Food Choice Motivations and Dietary Carbon Footprints in Adolescents: A Cross-Sectional Study
Source: Int J Environ Res Public Health. 2026 Mar 21;23(3):400. doi: 10.3390/ijerph23030400 (PMC13026819; doi:10.3390/ijerph23030400)
Supplement: Supplementary file 1 [file ijerph-23-00400-s001.zip › ijerph-4194873-supplementary.pdf]

Supplementary Materials

Table S1: Nutrition-related social media habits and perceptions of adolescents by gender.

Characteristics	Female (*n* = 131)	Male (*n* = 85)	Total (*n* = 216)	*p*-Value
Following nutrition-related pages on social media platforms				0.061
Yes	45 (69.2)	20 (30.8)	65 (30.1)	
No	86 (57.0)	65 (43.0)	151 (69.9)	
Nutrition-related videos appearing on Explore page				0.084
Yes	62 (66.0)	32 (34.0)	94 (43.5)	
No	14 (43.8)	18 (56.2)	32 (14.8)	
Sometimes	55 (61.1)	35 (38.9)	90 (41.7)	
Thinking that social media has an influence on food choice preferences				**<0.001**
Yes	72 (72.0)	28 (28.0)	100 (46.3)	
No	28 (40.0)	42 (60.0)	70 (32.4)	
Undecided	31 (67.4)	15 (32.6)	46 (21.3)	
The effect of food ads you see on social media on your hunger levels				**0.011**
Yes	58 (65.9)	30 (34.1)	88 (40.7)	
No	19 (41.3)	27 (58.7)	46 (21.3)	
Sometimes	54 (65.9)	28 (34.1)	82 (38.0)	
Following nutritionists on social networks				**0.036**
Yes	42 (71.2)	17 (28.8)	59 (27.3)	
No	89 (56.7)	68 (43.3)	157 (72.7)	
The reading of nutrition-related articles that you come across on social media				**<0.001**
Yes	70 (74.5)	24 (25.5)	94 (43.5)	
No	61 (50.0)	61 (50.0)	122 (56.5)	

Categorical variables are expressed as number (percentage). Chi-square test was used for categorical variables. Statistical significance is $p < 0.05$ and is indicated in bold.

Table S2: Relationship between carbon footprint per energy unit and food choice questionnaire scores in adolescents.

FCQ	R	*p*-Value
Health	**−0.173**	**0.011**
Mood	−0.025	0.719
Convenience	0.089	0.194
Sensory Appeal	−0.014	0.834
Natural Content	−0.099	0.149
Price	0.022	0.746
Weight Control	−0.095	0.166
Familiar	−0.033	0.631
Ethical Concern	−0.061	0.375
Total FCQ	−0.060	0.376

Spearman correlation analysis was applied. Statistical significance is $p < 0.05$ and is indicated in bold. Abbreviation: FCQ; Food Choice Questionnaire.
